# The Clinical Value of Blood miR-654-5p, miR-126, miR-10b, and miR-144 in the Diagnosis of Colorectal Cancer

**DOI:** 10.1155/2022/8225966

**Published:** 2022-10-13

**Authors:** Gang Du, Chongren Ren, Ju Wang, Jun Ma

**Affiliations:** ^1^General Surgery Department, Shanxi Bethune Hospital/General Surgery Department, Third Hospital of Shanxi Medical University, Taiyuan, Shanxi 030032, China; ^2^Tongji Hospital, Tongji Medical College, Huazhong University of Science and Technology, Wuhan, Hubei 430030, China

## Abstract

Colorectal cancer (CRC) is the third cause of cancer-related death and the fourth most frequently diagnosed cancer across the globe. The objective of this study is to obtain novel and effective diagnostic markers to enrich CRC diagnosis methods. Herein, exosomal miRNA expression data of CRC and normal blood were subjected to XGBoost algorithm, and 5 miRNAs related to CRC diagnosis were primarily confirmed. Then multilayer perceptron (MLP) classifiers were constructed based on different subsets. Via integrated feature selection (IFS), we noticed that the MLP classifier constructed by the first four miRNAs (miR-654-5p, miR-126, miR-10b, and miR-144) had the highest Matthews correlation coefficient (MCC). Subsequently, principal component analysis (PCA) for dimensionality reduction was performed on samples based on the miR-654-5p, miR-126, miR-10b, and miR-144 expression data. The signature based on these four feature miRNAs, as the analysis indicated, could effectively distinguish CRC samples from normal samples. Further, we extracted the exosomes from clinical blood samples and applied qRT-PCR analysis, which revealed that the expression of these four feature miRNAs was in the trend of that in the test set. Collectively, these four feature miRNAs might be tumor biomarkers in the serum, and our study offers innovative thinking on early-stage CRC diagnosis.

## 1. Introduction

As the third cause of cancer-related death and the fourth most frequently diagnosed cancer [1], colorectal cancer (CRC) presents a growing morbidity and death rate, making it a public health burden [2]. According to population and disease statistics, nearly 2.2 million new cases would be developed by 2030 [3]. CRC is a genotypically and phenotypically heterogeneous disease characterized by different molecular characteristics [4]. Accurate early diagnosis enables CRC patients to receive timely and precise treatment, thereby reducing CRC mortality. Although colonoscopy screening is the gold standard for CRC screening, its participation rate in population screening programs is still poor due to the invasive nature of the test and the need for adequate bowel preparation [5–8]. In addition, some studies have implied that carcinoembryonic antigen and calprotectin can be used as diagnostic markers for CRC, but their specificity and sensitivity are low, and they cannot be effectively applied to the early diagnosis of clinical CRC at present [9, 10]. Hence, it is necessary to develop effective biomarkers for CRC to improve the early diagnosis rate for CRC and offer effective biomarkers for CRC treatment.

Recently, exosome biomarkers containing multiple RNA and proteins have become the focus of research in cancer diagnosis and treatment [11]. Exosomes are tiny goblet vesicles with 30-140 nm in diameter that are secreted by cells including immune cells, neural cells, stem cells, and tumor cells [12–14]. Increasing research manifested that exosomes relate to tumorigenesis. Tumor-derived exosomes are involved in the exchange of genetic information between tumor cells and basal cells, thereby regulating angiogenesis and promoting tumor growth and invasion [15]. Currently, useful biomarkers have been identified from exosomes for the application in CRC diagnosis. It has been demonstrated that in blood exosomes, miR-125a-3p and miR-638 are helpful for early diagnosis of CRC in clinical practice [16, 17]. These all demonstrated the importance of exosomal miRNAs in screening early-stage CRC. Therefore, we further identified potentially effective exosomal miRNAs that may work for CRC diagnosis, so as their regulatory networks, which are beneficial for comprehensively understanding the molecular mechanisms underlying CRC development.

The rapid development of biotechnology in the age of big data stimulated the application of bioinformatics in medical research; bioinformatics technology based on high-throughput sequencing data is an effective and promising analytical tool for analyzing and identifying biomarkers for cancer diagnosis [18, 19]. Machine learning is a new artificial intelligence technique that has been gradually applied to medical research in recent years. Lian et al. [20] trained medulloblastoma stemness index based on a machine learning method of one-class logistic regression to obtain gene expression-based stemness index and methylation-based stemness index and further identified their corresponding potential drugs, which provides new ideas for the survival of medulloblastoma patients or targeting stem cells. Koppad et al. [21] screened diagnostic candidate genes for CRC based on six methods of machine learning classification including Adaboost, ExtraTrees, logistic regression, Naive Bayes classifier, random forest, and XGBoost. Thus, there is potential for wider application of novel bioinformatics methods to identify novel diagnostic biomarkers based on public databases.

In this study, by analyzing the miRNA expression data of CRC patients and normal people in the Gene Expression Omnibus (GEO) database, we preliminarily screened miRNAs with potential diagnostic value based on XGBoost and established a multilayer perceptron (MLP) classifier to determine the optimal miRNA combination by taking integrated feature selection (IFS). Thereafter, the clinical value of diagnostic markers in CRC was dissected by testing their levels in the blood exosomes of clinical patients with CRC. To conclude, our study provided potential biomarkers which are supposed to be effective to CRC clinical diagnosis.

## 2. Materials and Methods

### 2.1. Data Source and Preprocessing

Exosomal miRNA data of CRC patients and normal people were downloaded as GSE39833 (tumor: 88 and normal: 11) from Gene Expression Omnibus (GEO) (https://www.ncbi.nlm.nih.gov/geo/), annotated by the platform of Agilent-021827 Human miRNA Microarray G4470C GPL14767. Differential analysis was performed by R package “limma” [22] on the standardized miRNA expression data (|logFC| > 1.5, adjPvalue < 0.05).

### 2.2. XGBoost Feature Selection

XGBoost is a tree boosting scalable machine learning system, which generates a single strong learner by combining multiple weak learners. XGBoost estimates the value of the loss function through a second-order Taylor series and further reduces the likelihood of overfitting by applying regularization [23]. The objective function of XGBoost is a gradient advancing decision tree approach defined as

(1) $$\begin{array}{c} £\left(\phi \right)=\overset{n}{\underset{i=1}{\sum }}\text{l}\text{oss}\left(y_{i{\hat{y}}_{i}}\right)+\overset{k}{\underset{k=1}{\sum }}\Omega \left(f_{k}\right). \end{array}$$

Loss means training loss, *Ω*(*f*) represents the complexity of trees, and *k* stands for the amount of trees. The model can be optimized by minimizing the objective function. Hence, we adopted the addition training method to calculate the training loss and rapidly optimized the prediction of the *n*^th^ round of addition training by taking the Taylor expansion method. The optimal complexity of the tree was determined via the greedy algorithm.

In order to find miRNAs that could distinguish CRC from normal samples in GSE39833, we utilized XGBoost to rank the importance of feature miRNAs. Five characteristic miRNAs associated with CRC diagnosis were filtered for subsequent analysis. Then, based on SMOTE method, we applied python package “imblearn” and Bayesian optimization to resample the training set to reduce the effect caused by data disequilibrium.

### 2.3. Construction of the MLP Classifier

To construct a diagnostic classifier that was more precise, we constructed MLP classifiers of different subsets based on these five characteristic miRNAs by python package “sklearn” [24] after XGBoost feature selection. For the MLP classifier, hidden layers were set as 2, and all possible combinations were scanned in the first layer (the number of nodes from 1 to 5) and in the second layer (the number of nodes from 1 to 5) by sklearn.neural_network. Other parameters included (1) solver = ^“^adam^”^, (2) alpha = 0.001, (3) random_state = 1, and (4) max_iter = 1000.

### 2.4. Screen of Optimal Feature Genes

The MCC of the above classifiers was obtained using IFS. MCC is the correlation coefficient of binary classification between the observation and prediction, with its value between -1 and +1. +1 stands for a perfect prediction, while -1 for a total inconsistency between observation and prediction. The MCC value is a single score that is the most informative for the prediction quality of binary classifiers built in a confusion matrix environment [25]. The IFS curves were plotted, with abscissa for MLP classifiers based on different subsets and ordinate for MCC of subsets. The classifier with the highest MCC was selected as the optimal classifier for CRC diagnosis.

### 2.5. Principal Component Analysis (PCA)

PCA is a dimensionality reduction algorithm that is most widely adopted. Its main idea is to map the n-dimensional data in space onto the k-dimension, a novel orthogonal feature that is the principal component [26]. We performed PCA by the R package “FactoMineR” [27] based on the characteristic miRNA expression data in the optimal MLP classifier to explore the sample discriminatory capability of this classifier (https://www.rdocumentation.org/packages/FactoMineR/versions/2.4).

### 2.6. Clinical Collection of Blood Sample

Between 10-2018 and 10-2021, 100 patients with CRC and 120 healthy participants were recruited from Shanxi Bethune Hospital, Shanxi Academy of Medical Sciences, Tongji Shanxi Hospital, Third Hospital of Shanxi Medical University in Taiyuan city, Shanxi province, with their clinical information and serum samples collected (Supplementary Table 1). None of the CRC patients received any treatment, while their cancer stages were judged on the basis of the American Joint Committee on Cancer (AJCC) Cancer Staging Manual (7th Edition) [28]. Peripheral blood (5 ml) from all participants was collected in 5 ml clotting tubes (Greiner Bio-One, Austria). Serum was separated by centrifugation and stored at -80°C for subsequent miRNA extraction.

This research is approved by the Ethics Committee of Shanxi Bethune Hospital, Shanxi Academy of Medical Sciences, Tongji Shanxi Hospital, Third Hospital of Shanxi Medical University. Besides, all participants were well-informed about the necessary information of this study and signed the written informed consent.

### 2.7. Exosome Separation

The exosome separation followed the steps described by Han et al. [29]. And the exosomes acquired were resuspended in phosphate-buffered saline (PBS). The suspension was placed on a chloroform-coated copper grid with 0.125% Formvar and negatively stained with uranyl acetate. Morphological identification of the exosomes was by a transmission electron microscopy (TEM).

### 2.8. RNA Extraction and qRT-PCR

Total RNA from the obtained exosomes was extracted following the miRNeasy Micro Kit (QIAGEN, Germany), and RNA quantity and quality were tested via Agilent Bioanalyzer 2100 (Agilent, USA). cDNA was synthesized by reverse transcription from total RNA using SuperScript III Reverse Transcriptase kit (Invitrogen, USA), and qPCR was performed using SYBR Premix Ex Taq II (Takara, Japan). qRT-PCR was performed using ABI7500 (7500, ABI, USA), and the relative expression of all miRNAs was calculated using the 2^-*ΔΔ*CT^ method. U6 was the internal reference.  Table 1 shows primer sequences for feature miRNAs.

### 2.9. Statistical Analysis

Based on analysis performed by GraphPad 8.0, box plots were drawn. Differences in the relative expression of miRNAs between tumor and normal samples were analyzed using the *t*-test, and *p* < 0.05 indicated a difference that was statistically significant.

## 3. Results

### 3.1. Constructing the Diagnostic Model of CRC

**Figure 1:**
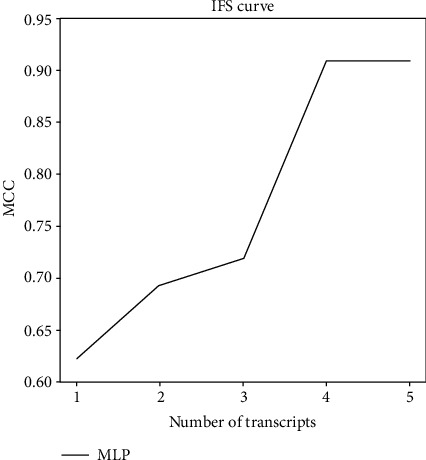
IFS curves of the five feature miRNAs based on MLP classifiers. Abscissa: the number of transcripts; ordinate: the MCC value.

56 differentially expressed miRNAs (DEmiRNAs) were obtained by normalization and differentially analyzing miRNAs data derived from CRC and normal exosomes. Subsequent XGBoost feature selection indicated the top five miRNAs with the best ability to distinguish sample types. To determine the optimal diagnostic classifier for CRC, we constructed different MLP classifiers and plotted IFS curves to visually select miRNA combinations. Through the IFS curve, it was found that the classification effect of the MLP classifier composed of the first four miRNAs (miR-654-5p, miR-126, miR-10b, and miR-144) was good, and the 10-fold cross-validation results showed that its MCC value was high (Figure 1), and the sensitivity of this model was 0.977, the specificity was 1.000, the accuracy was 0.980, and the MCC was 0.909.

### 3.2. Validation of the Performance of the Diagnostic Model

**Figure 2:**
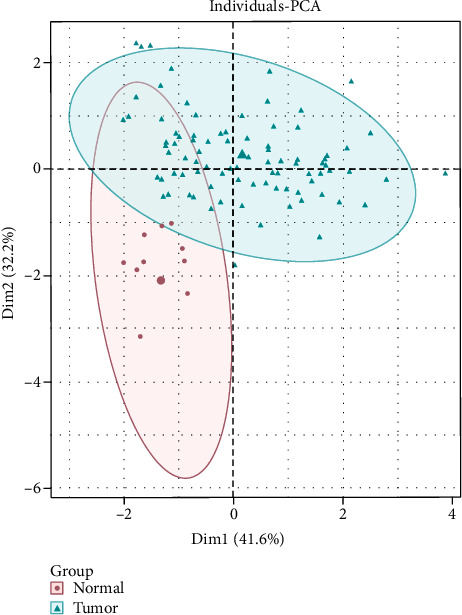
The PCA analysis for CRC and normal samples based on four feature miRNAs.

**Figure 3:**
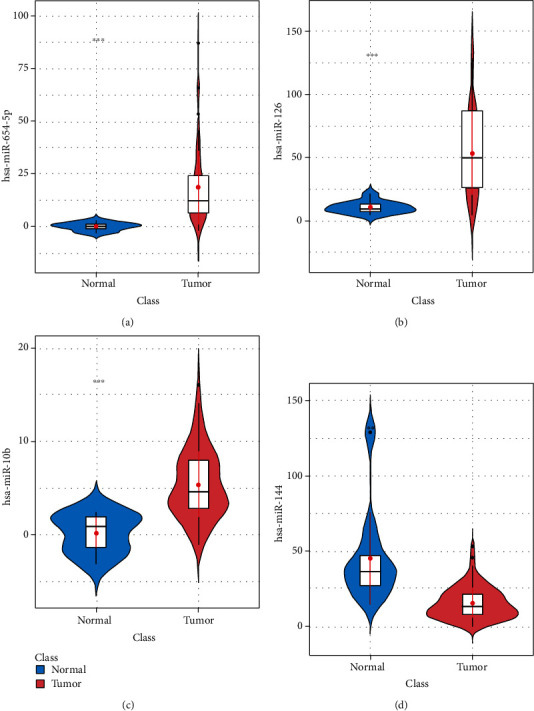
Violin plots of four feature miRNAs in the optimal MLP classifier. The violin plots of differentially expressed (a) miR-654-5p, (b) miR-126, (c) miR-10b, and (d) miR-144 in CRC and normal samples  ^∗∗∗^*p* < 0.001.

The expression data of four miRNAs in MLP classifiers in CRC and normal samples were subjected to PCA dimensionality reduction. Shown in Figure 2 were that PCA could significantly distinguish CRC and normal samples. Dim1 contributed 41.6% and Dim2 contributed 32.2%. From the violin plots, we could see that levels of blood exosomal miR-654-5p, miR-126, and miR-10b from CRC patients were markedly higher, but miR-144 was markedly lower than normal participants (Figures 3(a)–3(d)). The above results exhibited that the MLP formed by the former four miRNAs showed the value to assist CRC diagnosis.

### 3.3. qRT-PCR of miRNAs from Clinical Samples and Receiver Operator Characteristic (ROC) Analysis

**Figure 4:**
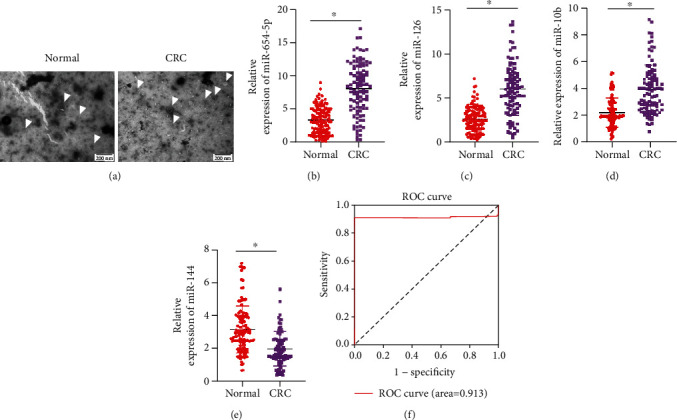
qRT-PCR and ROC curve analysis of miRNAs in clinical samples. (a) TEM observation of exosomes. Scale bar: 200 *μ*m. Box plots of differentially expressed (b) miR-654-5p, (c) miR-126, (d) miR-10b, and (e) miR-144 in CRC and normal samples. (f) ROC curves of 4-miRNA in clinical diagnosis  ^∗^*p* < 0.05.

To validate the performance of this model in clinical CRC diagnosis, we recruited 100 CRC and 120 healthy participants (Table 2), collected their blood samples, and extracted exosomes for qRT-PCR. Exosomes were first extracted from the blood of CRC patients as well as healthy participants, and the isolated exosomes were subsequently validated for size and morphology. Under a TEM, we could observe that the extracted exosomes were oval membrane-bound vesicles, which were about 50 nm-150 nm in diameter (Figure 4(a)). Thereafter, the qRT-PCR revealed that levels of blood exosomal miR-654-5p, miR-126, and miR-10b from CRC patients were markedly higher (Figures 4(b)–4(d)), but miR-144 was markedly lower than normal participants (Figure 4(e)). Data from qRT-PCR were collected for validation of the performance of the diagnostic model in CRC diagnosis. As results suggested, the ROC of the 4-miRNA diagnostic model was 0.913 (Figure 4(f)), and the recall of the model was 0.91, specificity was 0.34, accuracy was 0.6, and f1 was 0.67. Collectively, qRT-PCR on clinical samples validated that this 4-miRNA model could distinguish CRC and normal samples precisely, enabling these miRNAs to be biomarkers for CRC diagnosis.

## 4. Discussion

As key regulators in a variety of biological and physiological processes, miRNA dysregulation may be tightly linked to changes in the pathological environment of disease [30–32]. Colonoscopy is the gold standard for the pathological diagnosis of CRC, but it causes a large physical as well as psychological burden to patients due to its high invasiveness [5–8]. Owing to patients' avoidance of colonoscopy, CRC cannot be diagnosed promptly at the early stage and is only diagnosed at advanced stages when tumor metastasizes to other tissue [33]. The advantage of miRNA detection relative to invasive colonoscopy is that samples are more accessible in clinical practice both in body fluids and blood. At the same time, this noninvasive examination greatly alleviates the physical burden on patients [32, 34, 35]. Given its noninvasive and easily accessible properties, miRNAs are promising biomarkers in CRC diagnosis.

We here utilized XGBoost to determine the key features by ranking feature importance and recursive elimination. We determined the top 5 miRNAs that could accurately distinguish CRC cancer patients from healthy individuals and subsequently found via IFS method that the MLP classifier composed of the top four miRNAs was the best for CRC diagnosis. MLP is a dynamic classifier based on neural network, which could directly determine the separating hyperplanes between the two types of events, with high accuracy of classification and strong ability of parallel distribution processing. At present, there are also some studies on constructing CRC diagnostic classifiers based on machine learning algorithms. Koppad et al. [21] screened CRC diagnosis-related genes by the random forest algorithm, which has the advantage of avoiding data overfitting and reducing the computational load of the model. We aimed to filter biomarkers that could diagnose cancer. While MLP is to classify two types of events, therefore, it was our tool for identifying miRNAs that could assist CRC diagnosis.

The top four miRNAs selected by IFS (miR-654-5p, miR-126, miR-10b, and miR-144) could accurately diagnose CRC. These four miRNAs have all been reported in CRC. Reported by Li et al. [36], the decreased level of miR-654-5p is markedly correlated with the clinical stage of colon cancer by analyzing miR-654-5p level in tissue from CRC patients and normal participants, indicating that its level might be closely related to the CRC progression. As stated by Ebrahimi et al. [37], low miR-126 level in CRC is linked to CRC histological subtype, perineural tumor invasion, microsatellite instability pathological analysis, and lymph node distal metastasis. One study indicated that upregulated miR-10b is discovered in CRC patients with liver metastases, positively linked to advanced TNM stage, and able to predict advanced clinicopathological features and liver metastasis in CRC [38]. Research by Choi et al. [39] indicated that stool from CRC patients is a novel screening biomarker, and the miR-144 level in the stool has good sensitivity and specificity for CRC detection. Finally, we collected blood samples from CRC patients and normal participants for qRT-PCR, and the expression trends of miRNAs were consistent with those reported in the literature, which also validated the accuracy of our study. Further, PCA revealed that the MLP diagnostic classifier composed of miR-654-5p, miR-126, miR-10b, and miR-144 could well distinguish samples from CRC patients and normal individuals. Hence, these four miRNAs could be unique biomarkers for noninvasive examination of CRC.

However, limitations still exist. Our study utilized the limited numbers of public datasets and did not take into account factors like age, gender, ethnicity, and tumor TNM stages, which may affect miRNA expression. Hence, the construction of a more precise diagnosis model can be achieved by carrying a more detailed analysis on these factors, providing science-based evidence for the clinical noninvasive diagnosis of CRC. Overall, we performed XGBoost and constructed an MLP classifier to identify four miRNAs with the highest diagnostic value. PCA and ROC curves suggested favorable performance of the 4-miRNA classifier to distinguish CRC patients from normal individuals. This study sheds light on science-based theory for the noninvasive diagnosis of CRC.

## Figures and Tables

**Table 1:** Primers of feature miRNAs for qRT-PCR.

Gene ID	Forward sequence (5′-3′)	Reverse sequence (5′-3′)
miR-654-5p	GGGTGGTGGGCCGCAGAAC	CTCAACTGGTGTCGTGGA
miR-126	UCGUACCGUGAGUAAUAAUGCG	CAUUAUUACUUUUGGUACGCG
miR-10b	TACCCTGTAGAACCGAATTTGTG	CAGTGCGTGTCGTGGAGT
miR-144	TACAGTATAGATGAT	GTGCAGGGTCCGAGG
U6	ATTGGAACGATACAGAGAAGATT	GGAACGCTTCACGAATTTG

**Table 2:** Clinical information summary of 220 subjects.

	External validation cohort	
Variables	CRC (*n* = 100)	Normal (*n* = 120)
Gender		
Male	49 (49%)	51 (42.5%)
Female	51 (51%)	69 (57.5%)
Age		
<60	43 (43%)	47 (39.2%)
≥60	57 (57%)	73 (60.8%)
Tumor location		
Colon	44 (44%)	
Rectum	56 (56%)	
Stage		
1	24 (24%)	
2	17 (17%)	
3	30 (30%)	
4	29 (29%)	

## Data Availability

The data and materials in the current study are available from the corresponding author on reasonable request.

## Supplementary Materials

Supplementary Materials Supplementary Table 1: clinical information of 220 participants.

## References

[1] Goodarzi E., Beiranvand R., Naemi H., Momenabadi V., Khazaei Z. (2019). Worldwide incidence and mortality of colorectal cancer and human development index (HDI): an ecological study. *World Cancer Research Journal*.

[2] Arnold M., Sierra M. S., Laversanne M., Soerjomataram I., Jemal A., Bray F. (2017). Global patterns and trends in colorectal cancer incidence and mortality. *Gut*.

[3] Rawla P., Sunkara T., Barsouk A. (2019). Epidemiology of colorectal cancer: incidence, mortality, survival, and risk factors. *Gastroenterology Review*.

[4] Bogaert J., Prenen H. (2014). Molecular genetics of colorectal cancer. *Annals of Gastroenterology*.

[5] Strul H., Arber N. (2007). Screening techniques for prevention and early detection of colorectal cancer in the average-risk population. *Gastrointestinal Cancer Research*.

[6] Ciombor K. K., Wu C., Goldberg R. M. (2015). Recent therapeutic advances in the treatment of colorectal cancer. *Annual Review of Medicine*.

[7] Simon K. (2016). Colorectal cancer development and advances in screening. *Clinical Interventions in Aging*.

[8] U.S. Preventive Services Task Force (2002). Screening for colorectal cancer: recommendation and rationale. *Annals of Internal Medicine*.

[9] Vega P., Valentin F., Cubiella J. (2015). Colorectal cancer diagnosis: pitfalls and opportunities. *World Journal of Gastrointestinal Oncology*.

[10] von Roon A. C., Karamountzos L., Purkayastha S. (2007). Diagnostic precision of fecal calprotectin for inflammatory bowel disease and colorectal malignancy. *The American Journal of Gastroenterology*.

[11] Simpson R. J., Lim J. W., Moritz R. L., Mathivanan S. (2009). Exosomes: proteomic insights and diagnostic potential. *Expert Review of Proteomics*.

[12] Li X., Corbett A. L., Taatizadeh E. (2019). Challenges and opportunities in exosome research-perspectives from biology, engineering, and cancer therapy. *APL Bioengineering*.

[13] Hessvik N. P., Llorente A. (2018). Current knowledge on exosome biogenesis and release. *Cellular and Molecular Life Sciences*.

[14] Sedgwick A. E., D'Souza-Schorey C. (2018). The biology of extracellular microvesicles. *Traffic*.

[15] Hannafon B. N., Ding W. Q. (2013). Intercellular communication by exosome-derived microRNAs in cancer. *International Journal of Molecular Sciences*.

[16] Wang J., Yan F., Zhao Q. (2017). Circulating exosomal miR-125a-3p as a novel biomarker for early-stage colon cancer. *Scientific Reports*.

[17] Yan S., Dang G., Zhang X. (2017). Downregulation of circulating exosomal miR-638 predicts poor prognosis in colon cancer patients. *Oncotarget*.

[18] Gao C., Zhou C., Zhuang J. (2018). MicroRNA expression in cervical cancer: novel diagnostic and prognostic biomarkers. *Journal of Cellular Biochemistry*.

[19] Han Z., Li Y., Zhang J. (2020). Tumor-derived circulating exosomal miR-342-5p and miR-574-5p as promising diagnostic biomarkers for early-stage lung adenocarcinoma. *International Journal of Medical Sciences*.

[20] Lian H., Han Y. P., Zhang Y. C. (2019). Integrative analysis of gene expression and DNA methylation through one-class logistic regression machine learning identifies stemness features in medulloblastoma. *Molecular Oncology*.

[21] Koppad S., Basava A., Nash K., Gkoutos G. V., Acharjee A. (2022). Machine learning-based identification of colon cancer candidate diagnostics genes. *Biology*.

[22] Ritchie M. E., Phipson B., Wu D. (2015). limma powers differential expression analyses for RNA-sequencing and microarray studies. *Nucleic Acids Research*.

[23] Chen T., Guestrin C. XGBoost: a scalable tree boosting system.

[24] Yang F., Wang X., Ma H., Li J. (2021). Transformers-sklearn: a toolkit for medical language understanding with transformer-based models. *BMC Medical Informatics and Decision Making*.

[25] Chicco D., Jurman G. (2020). The advantages of the Matthews correlation coefficient (MCC) over F1 score and accuracy in binary classification evaluation. *BMC Genomics*.

[26] Ringner M. (2008). What is principal component analysis?. *Nature Biotechnology*.

[27] Garcia-Rudolph A., Garcia-Molina A., Opisso E., Tormos Munoz J. (2020). Personalized web-based cognitive rehabilitation treatments for patients with traumatic brain injury: cluster analysis. *JMIR Medical Informatics*.

[28] Edge S. B., Compton C. C. (2010). The American Joint Committee on Cancer: the 7th edition of the AJCC cancer staging manual and the future of TNM. *Annals of Surgical Oncology*.

[29] Han S., Li G., Jia M. (2021). Delivery of anti-miRNA-221 for colorectal carcinoma therapy using modified cord blood mesenchymal stem cells-derived exosomes. *Frontiers in Molecular Biosciences*.

[30] Treiber T., Treiber N., Meister G. (2019). Regulation of microRNA biogenesis and its crosstalk with other cellular pathways. *Nature Reviews. Molecular Cell Biology*.

[31] Bartel D. P. (2018). Metazoan microRNAs. *Cell*.

[32] Condrat C. E., Thompson D. C., Barbu M. G. (2020). miRNAs as biomarkers in disease: latest findings regarding their role in diagnosis and prognosis. *Cell*.

[33] Levine J. S., Ahnen D. J. (2006). Adenomatous polyps of the colon. *The New England Journal of Medicine*.

[34] Rupaimoole R., Slack F. J. (2017). MicroRNA therapeutics: towards a new era for the management of cancer and other diseases. *Nature Reviews. Drug Discovery*.

[35] Fesler A., Jiang J., Zhai H., Ju J. (2014). Circulating microRNA testing for the early diagnosis and follow-up of colorectal cancer patients. *Molecular Diagnosis & Therapy*.

[36] Li P., Cai J. X., Han F. (2020). Expression and significance of miR-654-5p and miR-376b-3p in patients with colon cancer. *World Journal of Gastrointestinal Oncology*.

[37] Ebrahimi F., Gopalan V., Wahab R., Lu C. T., Anthony Smith R., Lam A. K. Y. (2015). Deregulation of miR-126 expression in colorectal cancer pathogenesis and its clinical significance. *Experimental Cell Research*.

[38] Jiang H., Liu J., Chen Y., Ma C., Li B., Hao T. (2016). Up-regulation of mir-10b predicate advanced clinicopathological features and liver metastasis in colorectal cancer. *Cancer Medicine*.

[39] Choi H. H., Cho Y. S., Choi J. H., Kim H. K., Kim S. S., Chae H. S. (2019). Stool-based miR-92a and miR-144∗ as noninvasive biomarkers for colorectal cancer screening. *Oncology*.

