# Supplementary material for: The Clinical Value of Blood miR-654-5p, miR-126, miR-10b, and miR-144 in the Diagnosis of Colorectal Cancer
Source: Comput Math Methods Med. 2022 Oct 13;2022:8225966. doi: 10.1155/2022/8225966 (PMC9584656; doi:10.1155/2022/8225966)
Supplement: Supplementary Materials — Supplementary Table 1: clinical information of 220 participants. [file 8225966.f1.pdf]

sample ID	group	age	sex	stage	class	tumourlocation
2	test	79	male	1	CRC	colon
5	test	71	female	3	CRC	rectum
7	test	82	female	1	CRC	rectum
8	test	57	male	4	CRC	rectum
9	test	52	male	3	CRC	rectum
10	test	70	male	4	CRC	colon
12	test	55	male	3	CRC	colon
16	test	74	female	4	CRC	colon
17	test	58	male	3	CRC	rectum
18	test	62	female	3	CRC	rectum
19	test	59	female	4	CRC	rectum
21	test	53	female	3	CRC	rectum
24	test	58	female	2	CRC	rectum
26	test	60	female	2	CRC	colon
28	test	58	male	3	CRC	rectum
29	test	57	female	3	CRC	rectum
33	test	55	female	1	CRC	rectum
34	test	85	female	3	CRC	colon
35	test	53	female	4	CRC	rectum
36	test	64	male	4	CRC	colon
37	test	72	male	3	CRC	colon
39	test	57	male	4	CRC	rectum
43	test	68	female	3	CRC	rectum
45	test	64	male	2	CRC	rectum
47	test	63	male	2	CRC	colon
50	test	85	female	4	CRC	colon
52	test	59	male	4	CRC	colon
53	test	65	female	4	CRC	colon
55	test	89	male	4	CRC	colon
56	test	57	male	2	CRC	colon
59	test	70	male	4	CRC	rectum
61	test	59	male	3	CRC	colon
62	test	54	male	1	CRC	rectum
66	test	70	female	3	CRC	colon
67	test	80	female	3	CRC	rectum
68	test	55	female	4	CRC	rectum
70	test	55	female	2	CRC	colon

73	test	55	male	2	CRC	colon
75	test	82	male	1	CRC	rectum
76	test	78	female	4	CRC	colon
77	test	58	male	3	CRC	rectum
78	test	52	male	4	CRC	colon
79	test	64	male	3	CRC	rectum
82	test	73	female	4	CRC	colon
83	test	69	female	1	CRC	colon
85	test	68	female	3	CRC	rectum
86	test	59	female	1	CRC	colon
88	test	79	male	2	CRC	colon
92	test	57	male	3	CRC	rectum
94	test	55	female	2	CRC	rectum
96	test	59	male	1	CRC	rectum
97	test	59	female	4	CRC	rectum
100	test	72	female	1	CRC	rectum
103	test	72	female	3	CRC	rectum
105	test	71	female	4	CRC	colon
110	test	64	female	1	CRC	rectum
114	test	76	male	4	CRC	colon
116	test	55	male	2	CRC	colon
117	test	53	female	4	CRC	rectum
121	test	66	female	4	CRC	rectum
123	test	67	male	2	CRC	colon
127	test	58	male	1	CRC	rectum
128	test	65	male	4	CRC	colon
130	test	84	male	2	CRC	rectum
134	test	79	female	4	CRC	colon
136	test	90	male	4	CRC	colon
137	test	73	female	4	CRC	colon
139	test	57	male	1	CRC	colon
140	test	62	female	2	CRC	colon
145	test	88	female	3	CRC	rectum
147	test	82	female	2	CRC	rectum
150	test	81	female	2	CRC	rectum
152	test	84	female	1	CRC	rectum
155	test	57	female	1	CRC	colon
164	test	55	male	4	CRC	rectum

170	test	57	male	3	CRC	colon
172	test	57	female	1	CRC	rectum
175	test	59	male	3	CRC	rectum
176	test	54	female	1	CRC	colon
177	test	58	female	1	CRC	colon
179	test	57	male	4	CRC	colon
180	test	56	female	3	CRC	rectum
182	test	54	male	1	CRC	colon
183	test	63	female	3	CRC	rectum
184	test	72	male	4	CRC	colon
185	test	60	male	3	CRC	rectum
186	test	85	male	2	CRC	rectum
187	test	83	male	1	CRC	rectum
188	test	80	male	3	CRC	colon
189	test	87	female	4	CRC	colon
190	test	88	female	1	CRC	rectum
194	test	66	male	1	CRC	rectum
195	test	60	female	2	CRC	rectum
197	test	88	male	3	CRC	rectum
203	test	81	female	1	CRC	rectum
205	test	59	male	1	CRC	colon
209	test	52	female	3	CRC	rectum
214	test	90	female	1	CRC	rectum
218	test	67	male	3	CRC	rectum
220	test	56	male	3	CRC	rectum
